# Knowledge of palliative care and preference of end of life care: a cross-sectional survey of residents in the Chinese socio-cultural background of Macao

**DOI:** 10.1186/s12904-021-00798-z

**Published:** 2021-06-22

**Authors:** Kuai In Tam, Sok Leng Che, Mingxia Zhu, Sok Man Leong

**Affiliations:** Kiang Wu Nursing College of Macau, Est. Repouso No. 35, R/C, Macao SAR, China

**Keywords:** End of life care, Palliative care, Chinese culture, End of life care preferences, Life-sustaining treatments

## Abstract

**Background:**

Since the establishment of a hospice in the year 2000 and the development of a palliative care ward in 2019, there is no study examining public’s knowledge of palliative care, nor preference of end of life care in Macao.

**Aim:**

Targeting Chinese residents of Macao, the current study has 3 goals: i) to understand the level of knowledge of palliative care, ii) to explore the preference of end of life treatments, and iii) to identify the associated factors of the preference of end of life treatments.

**Methods:**

A cross-sectional questionnaire survey was conducted using a structured questionnaire. The study employed non-probability quota sampling through which Macao residents aged 18 and above were recruited between July and September 2020.

**Results:**

A total of 737 responses were valid. The average correct rate of palliative care knowledge ranged from 40.4% to 85.5%. Pertaining to end of life treatments, 62.0% of the respondents chose comfort care. However, almost half of the respondents agreed that life-sustaining treatments should not be stopped under any circumstances. Respondents who scored higher in palliative care knowledge and those with secondary and tertiary education were associated factors of choosing comfort care rather than life-sustaining treatments. In addition, respondents who agreed that futile life-sustaining treatments should be stopped were also associated with preference for comfort care.

**Conclusion:**

The understanding of palliative care amongst Macao residents is inadequate. Despite the public’s inclination towards comfort care, it is generally believed that life-sustaining treatments should not be stopped at the end of life. The study results suggest that not only the knowledge of palliative care should be enhanced amongst the general public in Macao, but information about life-sustaining treatments should also be offered to patients and families by healthcare professionals, in aiding end of life treatment decision making.

**Supplementary Information:**

The online version contains supplementary material available at 10.1186/s12904-021-00798-z.

## Background

Macao’s earliest implementation of palliative and end of life (EoL) care began when the first hospice was established in the year 2000. There has been very limited advancement since then, and the latest being the establishment of a palliative care ward within the government hospital in 2019, both the hospice and the palliative care ward are fully funded by the Macao government. While there is a limited extent of home visit offered by the hospice, the main focus of palliative care in Macao has remained on the inpatient service.

With respect to government policy, the Macao government formulated a ten-year action plan to develop and improve elderly care services in 2016, and the action plan emphasised the expansion of the existing palliative and EoL care services as well as the development of EoL care services in nursing homes [1]. Regarding legislations on EoL issues, there is no advance directives legislation in Macao. Patients and their families can decide to sign an agreement stating the decision to reject resuscitation or other life-sustaining treatments (LST). However, the agreement is based primarily on hospital’s internal policy whose nature is conceptually different from advance directives, and the agreement is not legally binding.

In terms of knowledge provision, the ten-year plan has made clear goals in promoting life and death education amongst the public, and enhancing the knowledge level of palliative and EoL care of the healthcare workforce [1]. Whilst it is important to improve palliative and EoL care services, it is equally important to raise people’s awareness and understanding about such care. Although public education of life and death issues was included as part of the short-term goals between 2016 and 2017, there has not been any official review evaluating the actual outcomes of the ten-year action plan in Macao. This situation is worth noting, especially when the lack of knowledge was found to be the key impediments to the overall development and acceptance of palliative and EoL care across different regions [2–9].

Literature in the subject of public knowledge of palliative and EoL care is found to be relatively limited; there are only a handful of studies that have conducted this type of research in specific locations, including Northern Ireland, Ireland, Sweden, Italy, Australia, Scotland, the United States, Hong Kong and Japan [2–7, 10–13]. Most of the available studies revealed that knowledge of palliative and EoL care amongst the general population is inadequate. Temel et al. [12]’s study found that around 70% of the US respondents do not have adequate knowledge about palliative care; Westerlund et al. [4] and Hirai et al. [3] shared similar findings in which respondents showed limited understanding of palliative and EoL care. Amongst Chinese population, recent studies conducted in Hangzhou and Hong Kong also found that Chinese respondents have little understanding about palliative and EoL care [14, 15].

In Macao, there has been some, though limited, research effort in exploring how people in Macao perceived EoL decision making. A study by Ho and Sanders [16] found that older Chinese in Macao tended to opt for institutionalised care at the EoL as they did not want to be a burden to family. However, Ho and Sanders [16]’s study was not focused on the preference relating to EoL care, rather, its primary focus was on EoL decision making amongst older Chinese in Macao. Nevertheless, Ho and Sanders [16]’s study was the first of its kind to explore people’s thoughts and decision making about EoL issues in Macao.

Taking into consideration adequate understanding of palliative and EoL care was shown to encourage terminally ill patients to choose EoL care, and thereby improve their quality of life at the end of their lives [12], this research aimed to fill this knowledge gap by exploring the public’s knowledge of palliative care, and their preferences regarding EoL care within the Chinese socio-cultural environment of Macao. More significantly, our study aimed to investigate elements that could potentially influence people’s decision between choosing palliative care or LST.

## Methods

### Research design

The study was a cross-sectional survey using a structured questionnaire.

### Sample

The study employed non-probability quota sampling according to the demographic statistics at the end of 2019 in Macao [17]. Macao residents aged eighteen and above, and are able to sufficiently understand informed consent materials and questionnaire content were recruited. Exclusion criteria for this study included: i) Individuals who have severe hearing impairment and individuals who are unable to communicate, and ii) Individuals who may feel their levels of distress may hinder capacity for informed consent and/or consider participation overwhelming.

The sample size for this cross-sectional survey study was calculated according to Charan & Biswas [18] and Pourhoseingholi et al. [19]’s sample size calculation. The significance criterion in this study was set to 0.5, with the absolute error of 5% and at type 1 error of 5%. The study aimed to recruit at least 384 respondents.

### Recruitment

In applying the quota sampling, the study advertised and recruited respondents from tertiary education institutions, social media platforms and two of the largest social organisations for adults with different backgrounds across different age groups; self-administered electronic version of the questionnaire was completed through the online platform Survey Monkey (https://www.surveymonkey.com/). The Internet Protocol (I.P.) address of respondents was the only linked identifier which made the survey partly anonymised. For individuals aged 60 and above, recruitment was conducted through day centres for older adults, and the questionnaire was completed face-to-face by trained interviewers. Data was collected between July and September 2020.

### Questionnaire

Having reviewed a number of similar studies, the study referenced the questionnaires developed by McIlfatrick et al. [6], Chung et al. [7] and Hsu et al. [20] enquiring Macao people’s perceptions of palliative and EoL care. The questionnaire was assessed by three experts in the field of palliative and EoL care from Beijing, Macao and Hong Kong. The questionnaire was revised according to the experts’ comments. The final questionnaire included three sections.

The first section collected the sociodemographic data of respondents, including age, gender, education level, marital status, religious beliefs, employment status, occupation and average monthly income in the past year. The second section focused on the knowledge of palliative care, enquiring respondents whether they have heard of palliative care and inviting them to choose from the following options “I have heard about it and know it very well”, “I have heard about it but don’t know it well”, “I have not heard of it before but want to know more”, or “I have not heard of it before and I don’t want to know more”. Moreover, in this section, a 9-item scale was structured after adopting the questions from existing literature [6, 7, 20] and took into considerations the sociocultural conditions of Macao. The 9-item scale was devised to understand respondents’ knowledge regarding the goal and service of palliative care, and the reliability of the scale was checked in the pilot stage, with the Item-Content Validity Index of 0.97 and the Cronbach’s α of 0.75, indicating that the scale was primarily suitable to enquire people’s knowledge of palliative care.

In the third section, respondents’ preference towards EoL treatment options was explored with two questions. The first question asked: “If the doctor has diagnosed you with an incurable illness, and you are estimated to have less than 6 months to live, which of the following would you choose?” Respondents were presented with 4 options: i) “I will accept all life prolonging treatments, even though discomfort or suffering may occur during the treatment process”, ii) “I will accept treatments that can ease pain and suffering, or alleviate discomfort caused by symptoms, even though my life may not be extended”, iii) “I don’t know/ I don’t have a decision”, iv) “I don’t want to answer”. The second question asked: “If the doctor has diagnosed you with an incurable illness, and you are estimated to have less than 6 months to live, would you agree that all LST should not be stopped under any circumstances?”, respondents were then presented with 5 statements: i) strongly agree, ii) agree, iii) neither agree nor disagree, vi) disagree and v) strongly disagree.

### Ethics

The study has obtained ethical approval from Kiang Wu Nursing College of Macau. All methods were employed in accordance with relevant guidelines and regulations. Respondents were asked to give their consent prior to the commencement of the questionnaire survey. Respondents were explained that they could stop or withdraw from the survey any time.

### Statistical analysis

Raw data were initially revised and coded in Microsoft Office Excel 2013. The study utilised Statistical Package for the Social Sciences Version 22 (SPSS, v22) for data manipulation and subsequent analysis. Descriptive analyses were performed on the collected demographic data, knowledge level of palliative care and attitude towards EoL care. To determine associated factors of palliative care as the preferred form of EoL care, logistic regression models were performed, adjusting for demographic covariates and knowledge level of palliative care, with estimated odds ratio (OR) with their 95% confidence intervals. The threshold for statistical significance was set to p < 0.05. Only respondents who completed the full questionnaire were considered valid for logistic regression. The management of research data and the process of study was performed in full compliance with the requirements stipulated in the internal regulations of the Research Management and Development Department of Kiang Wu Nursing College of Macau.

## Results

### Demographic characteristics of study respondents

The study received a total of 1001 responses in which 737 were valid, the completion rate was 73.6%. Amongst all respondents, 65.0% of them were female, aged between 19 and 101; 66.5% of respondents were married or cohabited, and 20.9% of respondents were not married; 56.9% of the respondents had college level or higher; 24.2% of respondents were working as professionals and 11.7% were medical (assistant) professionals. Owing to the dominant hospitality and gaming industry of Macao, 18.0% of respondents were attendants, 38.5% of respondents had cared for relatives or friends suffering from terminal illness (Table 1).

**Table 1:** Demographic characteristics of respondents (*N* = 737)

Variables	n	%
**Gender**		
Male	258	35.0
Female	479	65.0
**Age**		
18–39	310	42.1
40–64	305	41.4
≥ 65	122	16.6
**Education**		
Primary school or below	96	13.0
Secondary school	216	29.3
Bachelor or above	419	56.9
Other	6	0.8
**Marital status**		
Not married	154	20.9
Married/ cohabited	490	66.5
Separated/ divorced	38	5.2
Widowed	55	7.5
**Children**		
Yes	519	70.4
No	218	29.6
**Religious belief**		
None	428	58.1
Christianity	121	16.4
Buddhism/ Chinese folk beliefs	188	25.5
**Occupation**		
Professional	178	24.2
Medical (assistant) professional	86	11.7
Technician	108	14.7
Attendant	133	18.0
Disciplined services/ other	41	5.6
Not employed^a^	191	25.9
**Average monthly income in the past year**		
< 9,999	149	20.2
10,000 ~ 19,999	141	19.1
20,000 ~ 29,999	177	24.0
≧30,000	224	30.4
No answer	46	6.2
**Cared for relatives or friends suffering from terminal illness**		
Yes	284	38.5
No	453	61.5
**Self-rated health**		
Good	305	41.4
Not good	432	58.6

*Note*: Due to rounding, percentages may not always appear to add up to 100%

^a^Including student, unemployed, housewife/ househusband and retired

### Respondents’ knowledge of palliative care

Over half of the study respondents had heard of the term ‘palliative care’, however, less than 10% of them identified themselves to be very knowledgeable about it. There was still over 40% of study respondents who had no awareness about palliative care (Table 2).

**Table 2:** Respondents’ awareness of palliative care (*N* = 737)

	**n**	**%**
Have you heard of palliative care, and how would you describe your level of knowledge of palliative care?		
Yes, and very knowledgeable	71	9.6
Yes, but not very knowledgeable	337	45.7
No, but would like to know more	295	40.0
No, and would not like to know	34	4.6

Regarding the understanding of palliative care, the average correct rate of palliative care knowledge was 62.9% (ranging from 40.4% to 85.5%). The majority of respondents (85.5%) understood that the goal of palliative care is to eliminate pain and suffering for patients. Respondents were also able to correctly identify that palliative care satisfies patients’ psychological and spiritual needs, and palliative care takes care of patients as well as the need of families. However, almost half of the respondents identified that palliative care is only suitable for terminal cancer patients, and a significant number of respondents (72.3%) supported the misconception that palliative care offers no treatment to patients, receiving palliative care equals to waiting for their death (Table 3).

**Table 3:** Knowledge of palliative care amongst respondents (*N* = 737)

	**n**	**%**	**Ranking**
A. Palliative care is only suitable for terminal cancer patients^a^	351	47.6	8
B. Palliative care takes care of patients as well as the need of families	590	80.1	3
C. One can only receive palliative care at the Hospice and Palliative Centre^a^	298	40.4	9
D. Patients can still receive palliative care in normal hospital wards	379	51.4	6
E. While patients are receiving routine treatments, they can still receive palliative care	443	60.1	5
F. If one receives palliative care, it means healthcare professionals will not give any treatments to the patients, and letting the patients to “wait for their death”^a^	533	72.3	4
G. The purpose of palliative care is to cure patients’ illnesses^a^	359	48.7	7
H. The purpose of palliative care is to relieve or eliminate patients’ suffering	630	85.5	1
I. To satisfy patients’ psychological and spiritual needs is part of palliative care	591	80.2	2
**Average correct rate**		**62.9**	

^a^Reverse coded item

### Attitudes towards end of life treatment options

Table 4 indicates that over half of the study respondents (62.0%) chose treatments that would alleviate their suffering rather than life-prolonging treatments, even though their limited life might not be extended. Under one-fifth of respondents (18.6%) had wished to have their lives prolonged at all cost, even though suffering or discomfort might occur during this process. There was also almost one-fifth of respondents who did not know or did not have a decision.

**Table 4:** Attitudes towards end of life treatment options (*N* = 737)

	**n**	**%**
If the doctor has diagnosed you with an incurable illness, and you are estimated to have less than 6 months to live, which of the following would you choose?		
I will accept all life-prolonging treatments, even though discomfort or suffering may occur during the treatment process	137	18.6
I will accept treatments that can ease pain and suffering, or alleviate discomfort caused by symptoms, even though my life may not be extended	457	62.0
I don’t know/I don’t have a decision	135	18.3
I don’t want to answer	8	1.1

### Attitudes towards life-sustaining treatments

As shown in Table 5, when respondents were asked if they would agree that LST should not be stopped under any circumstances, almost half (44.9%) of the respondents agreed and strongly agreed. Only a third of the study respondents disagreed and strongly disagreed with the statement (33.4%). This result is significant in that it indicates conflicting wishes amongst study respondents in Macao regarding EoL treatment options. Whilst the above results show that over half of the study respondents opted for comfort care at the end of their lives, a significant portion of them also decided that life should be sustained under any circumstances.

**Table 5:** Attitude towards life-sustaining treatments (*N* = 737)

	**n**	**%**
If the doctor has diagnosed you with an incurable illness, and you are estimated to have less than 6 months to live, would you agree that all life-sustaining treatments should not be stopped under any circumstances?		
Strongly agree	114	15.5
Agree	217	29.4
Neither agree nor disagree	96	13.0
Disagree	210	28.5
Strongly disagree	36	4.9
I don’t know/ Don’t want to answer	64	8.7

### Factors associated with end of life care preferences

The level of education amongst respondents was found to influence the preference of EoL care; respondents who had attained secondary education were 2.87 times (aOR = 2.87, *p* = 0.04) more likely to prefer comfort care, while respondents who had undergraduate degree or above were almost 4 times (aOR = 3.68, *p* = 0.01) more likely to choose comfort care at the EoL when comparing with those with primary education. In addition, knowledge level of palliative care affected EoL treatment options; for every score increased in knowledge level, respondents had 8% higher odds of choosing comfort care (*p* = 0.04). Regarding attitude towards LST, respondents who did not agree with “LST should not be stopped” were 3 times (aOR = 3.27, *p* < 0.001) more likely to prefer comfort care when comparing with respondents who agreed with LST at the EoL. All other factors were not significantly correlated with EoL treatment options (Table 6).

**Table 6:** Logistic regression models of palliative care as the preferred form of EoL care (*N* = 632)

Variables	*B*	*SE*	Wald	*P*-value	Exp (*B*)	95% CI for Exp (*B*)	
						Lower	Upper
**Constant**	-2.09	0.75	7.68	0.01	0.12		
**Female (ref.: male)**	0.23	0.20	1.28	0.26	1.26	0.84	1.88
**Age (ref.:18–39 years)**							
40–64 years	0.21	0.23	0.89	0.35	1.24	0.80	1.93
≧65 years	0.74	0.54	1.88	0.17	2.09	0.73	6.00
**Education (ref.: primary school or below)**							
Secondary school	1.05	0.52	4.08	0.04	2.87	1.03	8.00
Bachelor or above	1.30	0.57	5.22	0.02	3.68	1.20	11.28
**Marital status (ref. unmarried)**							
Married/ cohabited	-0.32	0.35	0.87	0.35	0.72	0.37	1.43
Separated/ divorced	0.69	0.59	1.35	0.25	1.99	0.62	6.39
Widowed	0.34	0.53	0.40	0.53	1.40	0.49	3.97
**Children (ref.: none)**	0.02	0.31	0.00	0.95	1.02	0.55	1.87
**Religious belief (ref.: none)**							
Christianity	-0.31	0.26	1.37	0.24	0.74	0.44	1.23
Buddhist/ Chinese folk beliefs	0.38	0.23	2.85	0.09	1.47	0.94	2.29
**Occupation (ref.: Disciplined services/ other)**							
Professional	0.13	0.39	0.11	0.74	1.14	0.53	2.46
Medical (assistant) professional	0.72	0.48	2.28	0.13	2.05	0.81	5.21
Technician	0.23	0.43	0.29	0.59	1.26	0.54	2.93
Attendant	0.22	0.43	0.25	0.62	1.24	0.53	2.89
Not employed	0.38	0.47	0.65	0.42	1.46	0.58	3.65
**Average monthly income in the past year (ref.: < 9,999)**							
10,000–19,999	0.06	0.39	0.02	0.87	1.06	0.49	2.29
20,000–29,999	-0.05	0.39	0.02	0.89	0.95	0.45	2.02
≧30,000	-0.31	0.39	0.62	0.43	0.73	0.34	1.58
**Cared for relatives/ friends (ref.: no)**	0.27	0.19	2.10	0.15	1.31	0.91	1.88
**Good Self-rated health (ref.: not good)**	0.17	0.19	0.78	0.38	1.18	0.81	1.72
**Palliative care knowledge scores**	0.08	0.04	4.40	0.04	1.08	1.01	1.16
**Attitude towards life-sustaining treatments (ref.: agree)**	1.18	0.18	41.43	0.00	3.27	2.28	4.69

## Discussion

Our study is, to our knowledge, the first population-based survey investigating residents’ preference of EoL treatment options in Macao. The current study has established a reliable examination enquiring residents’ preference as well as identifying factors influencing such preference.

Macao has over 20 years of history of providing palliative and EoL care, however, revealed in this study is the lack of understanding people have regarding palliative care. Particularly, respondents believed palliative care is only for people with terminal cancer (see Table 3), and a large number of respondents perceived palliative care as a pessimistic way to wait for death (see Table 3). Results of this study share similarity with a study based in Hangzhou and Hong Kong, in that the majority of study respondents had not heard of palliative care [13–15]. The insufficient understanding regarding palliative and EoL care is also found in a number of other studies across different contexts such as Japan, China, Scotland, the United Kingdom and Italy [3, 5, 21–23].

Although the Chinese taboo relating to death and dying has cast a significant barrier in promoting palliative and EoL care, our study shows that over 62.0% of respondents in Macao preferred palliative care knowing that their lives might not be prolonged. The acceptance of palliative care found in our study also reflects in the Hangzhou study mentioned earlier [14]. Nevertheless, Macao residents consciously chose palliative care over LST with potential suffering, a significant number of residents still agreed that LST should never be stopped under any circumstances. The contradictory phenomenon is also observed in Taiwan wherein palliative care is relatively well established. Ranking the first amongst Asian regions, the 2015 Quality of Death index indicated that Taiwan has one of the best palliative and EoL care in the world [24]. Despite the fact that Taiwan has a dependable palliative care foundation, healthcare professionals continue to struggle with ethically challenged decisions in caring for terminal patients, such as the provision and withdrawal of LST for terminal patients [25, 26]. On the other hand, owing to Macao’s limited palliative care development since its first introduction, a significant number of respondents in this study still agreed that LST, as a clinical norm [27], should not be stopped under any circumstances, even though palliative care would have been their preferred EoL option. Besides, previous studies in Hong Kong found that people’s understanding regarding LST was inadequate [15, 28, 29], and this finding may lend support to the conflicting wishes observed amongst the respondents of this study, indicating the public of Macao may also lack sufficient understanding regarding LST. Hence, the findings of our study reflect a need for public education on palliative care as well as LST, enabling people to better understand different types of care and treatments available, as well as the best suitable treatments for them at the end of their lives.

### Limitations

The study has employed quota sampling to recruit sample respondents who would meet the demographic characteristics of the public of Macao, but the non-probability sampling was still a limitation. In addition, the study primarily collected data by way of online questionnaire survey, which may be related to the higher education tendency of participants in this study; both conditions could elicit selection bias in this study.

## Conclusions

The current study found that people’s understanding of palliative care is inadequate in Macao. Although the public of Macao gravitated towards comfort care over futile life prolongation, there was still a significant number of people who did not want LST to stop at the end of their lives. This conflicting treatment decision is, proposed by this study, not only related to the lack of understanding regarding palliative and EoL care, but also related to the insufficient knowledge about the specificities of LST offered in acute hospital setting. Being the first study in Macao to target the general population, results of this study suggest that there is a need to strengthen public education with respect to palliative care, EoL care and LST. In addition, it is equally important to encourage professional education on palliative care, in pursuit of equipping healthcare workers to support better and more appropriate end of life treatment decision making amongst patients and families.

## Supplementary Information

**Additional file 1**

## Data Availability

Raw data may be obtained from the corresponding author on reasonable request.

## Abbreviations

EoL: End of life
LST: Life-sustaining treatments
