# Supplementary material for: Knowledge of palliative care and preference of end of life care: a cross-sectional survey of residents in the Chinese socio-cultural background of Macao
Source: BMC Palliat Care. 2021 Jun 22;20:87. doi: 10.1186/s12904-021-00798-z (PMC8220704; doi:10.1186/s12904-021-00798-z)
Supplement: Supplementary file 1 — Additional file 1 [file 12904_2021_798_MOESM1_ESM.docx]

**Knowledge of palliative care and preference of end of life care: A cross-sectional survey of residents in the Chinese socio-cultural background of Macao**

**Questionnaire**

Are you a resident of Macao (Macao ID card holder)?

□ Yes. Please answer the next question.

□ No. The questionnaire has been completed. Thank you.

Please answer the following regarding your understanding of the goal and content of this research, and your willingness to participate in this research.

□ I understand clearly the goal and content of this research and I am willing to participate in this research by filling in this questionnaire.

□ I do not want to participate, thank you.

If you have any question regarding this research and this questionnaire, please contact our team: 62439428.

After you have completed the questionnaire, if you have any question, please contact our principle investigator Professor Leong Sok Man; Tel: 82956207; E-mail: lsm@kwnc.edu.mo.

If you have any suggestions or complaints, please contact the Research Management & Development Department at Kiang Wu Nursing College of Macau. (Tel: 8295 6206)

# Knowledge of Palliative Care

## 1. Have you ever heard of palliative care?

| □ I have heard about it and know it very well |
| --- |
| □ I have heard about it but don’t know it well |
| □ I have not heard of it before and I don’t want to know more |
| □ I have not heard of it before and I don’t want to know more |

## 2. Which description of palliative care do you think is correct? Please choose the most suitable answer according to your understanding.

| **Item** | **Yes** | **No** | **Don’t know** |
| --- | --- | --- | --- |
| 1. Palliative care is only suitable for terminal cancer patients | 1 | 2 | 3 |
| 1. Palliative care takes care of patients as well as the need of families | 1 | 2 | 3 |
| 1. One can only receive palliative care at the Hospice and Palliative Centre | 1 | 2 | 3 |
| 1. Patients can still receive palliative care in normal hospital wards | 1 | 2 | 3 |
| 1. While patients are receiving routine treatments, they can still receive palliative care | 1 | 2 | 3 |
| 1. If one receives palliative care, it means healthcare professionals will not give any treatments to the patients, and letting the patients to "wait for their death" | 1 | 2 | 3 |
| 1. The purpose of palliative care is to cure patients' illnesses | 1 | 2 | 3 |
| 1. The purpose of palliative care is to relieve or eliminate patients' suffering | 1 | 2 | 3 |
| 1. To satisfy patients‘ psychological and spiritual needs is part of palliative care | 1 | 2 | 3 |

# Attitude towards End-of-Life care

## **If the doctor has diagnosed you with an incurable illness, and you are estimated to have less than 6 months to live, which of the followings would you choose**?

□ I will accept all life prolonging interventions, despite discomfort or sufferings may occur during the treatment process

□ I will accept treatment interventions that can ease pain and suffering, or alleviate discomfort caused by symptoms, despite my life may not be extended

□ I don’t know/ I don’t have a decision

□ Don’t want to answer

1. **If the doctor has diagnosed you with an incurable illness, and you are estimated to have less than 6 months to live, would you agree that all life-sustaining interventions should not be stopped under any circumstances?**

| □ Strongly agree | □ Agree | □ Neither agree nor disagree | □ Disagree | □ Strongly disagree |
| --- | --- | --- | --- | --- |
| □ I don’t know/ don’t want to answer | |  |  |  |

# Personal Information

| 1. Have you ever cared for relatives or friends suffering from terminal illness? | | | | | | | | | | □Yes | | | | | □No | | | | | | |
| --- | --- | --- | --- | --- | --- | --- | --- | --- | --- | --- | --- | --- | --- | --- | --- | --- | --- | --- | --- | --- | --- |
| 2. How would you rate your overall health now? | | | | | | | | | | | | | | | | | | | | | |
|  | | □Very poor | □Poor | | | □Fair | | | | | □Good | | | | | | | | □Excellent | | |
| 3. Gender | | | | | □Male | | | □Female | | | | | | | | | | | | | |
| 4. Age | | | | | ____________years-old | | | | | | | | | | | | | | | | |
| 5. Education level | | | | | □No formal education | | | □Primary school or below | | | | | | | | □Junior high school | | | | □High school | |
|  | | | | | □Bachelor | | | □Master's degree or above | | | | | | | | □ Other | | | | | |
| 6. Marital status | | | | | □Not married | | □Married/ cohabited | | | | | | | □Separated/ divorced | | | | | | | □Widowed |
| 7. How many children do you have? | | | | | | | | | | | | _________ | | | | | | | | | |
| 8. Do you have any religious beliefs? | | | | | □ No (Go to question 9) | | | | | □ Yes (Go to question 8.1) | | | | | | | | | | | |
|  | | 8.1 Your religion is | | | □ Catholicism | | | | | □ Christian | | | | | | | | □ Buddhism | | | |
|  | |  | | | □ Chinese folk beliefs | | | | | □ Other | | | | | | | | | | | |
| 9. Your current employment status is | | | | | □ Employed (Go to question 10) | | | | | | | | □ Self-employed (Go to question 10) | | | | | | | | |
|  | | | | | □ Student (Go to question 11) | | | | | | | | □ Unemployed (Go to question 11) | | | | | | | | |
|  | | | | | □ Retired (Go to question 11) | | | | | | | | □ Housewife/ househusband (Go to question 11) | | | | | | | | |
| 10. Your current occupation is | | | | | | | | | | | | | | | | | | | | | |
|  | □ Manager | | | | | | | | | | | | | | | | | | | | |
|  | □ Professional | | | | | | | | | | | | | | | | | | | | |
|  | □ Medical professional | | | | | | | | | | | | | | | | | | | | |
|  | □ Medical assistant professional | | | | | | | | | | | | | | | | | | | | |
|  | □ Sports/ artistic technician | | | | | | | | | | | | | | | | | | | | |
|  | □ Administrative technician | | | | | | | | | | | | | | | | | | | | |
|  | □ Disciplined services | | | | | | | | | | | | | | | | | | | | |
|  | □ Personal service worker | | | | | | | | | | | | | | | | | | | | |
|  | □ Affairs supporting staff | | | | | | | | | | | | | | | | | | | | |
|  | □ Gaming attendant | | | | | | | | | | | | | | | | | | | | |
|  | □ Attendant | | | | | | | | | | | | | | | | | | | | |
|  | □ Automotive mechanic | | | | | | | | | | | | | | | | | | | | |
|  | □ Entry-Level Mechanic | | | | | | | | | | | | | | | | | | | | |
|  | □ Other： | | | | | | | | | | | | | | | | | | | | |
| 11. What is your average monthly income in the past year (patacas)? | | | | | | | | | | | | | | | | | | | | | |
|  | | □ <5,000 | | □ 5,000~9,999 | | | | | □ 10,000~19,999 | | | | | | | | □ 20,000~29,999 | | | | |
|  | | □ 30,000~39,999 | | □ 40,000~59,999 | | | | | □ ≥60,000 | | | | | | | | □ Don’t want to answer | | | | |

**》》》》》》》》》》》》》》》》》》》》》》》》》》End of questionnaire. Thank you.《《《《《《《《《《《《《《《《《《《《《《《《《《《**
